# Atypical Chest Wall Cystic Hygroma in a Toddler: A Case Report and Comprehensive Review

**DOI:** 10.7759/cureus.98881

**Published:** 2025-12-10

**Authors:** Ayed S Askar, Shrouk F Mohamed, Ahmed Taha, Muhammad Obeydan, Safi Nassan

**Affiliations:** 1 Surgery, Idlib University Hospital, Idlib, SYR; 2 Medicine and Surgery, Faculty of Medicine, Alexandria, EGY; 3 Pediatrics, Idlib University, Idlib, SYR; 4 Pediatric Surgery, King Abdulaziz Medical City, Jeddah, SAU

**Keywords:** breast, chest wall tumor, lymphangioma, macro-cystic hygroma, young child

## Abstract

Lymphangiomas, also known as cystic hygromas, are rare benign lymphatic malformations that typically occur in the head and neck region. Breast or chest wall involvement in pediatric patients is exceptionally rare, with only a few cases reported in the literature. We report a case of a three-year-old boy who presented with a progressively enlarging right breast mass. Unsupervised topical creams and massage led to further enlargement. Laboratory investigations showed mild elevation in prolactin levels, which normalized after surgery. Ultrasonography demonstrated multiple cystic lesions, the largest of which was oval and measured 26 mm in diameter, along with an enlarged right axillary lymph node. Computed tomography (CT) scan showed a well-defined oval mass (51 × 26 mm) in the right chest wall. The patient underwent surgical excision, and a complete histopathological examination was performed. Histopathological examination revealed multiple lymphatic spaces lined by flattened endothelial cells forming a tumorous mass, with both cavernous and capillary-type lumens. The diagnosis of lymphangioma was confirmed. No mammary tissue was detected, and cancer was excluded. Subsequent evaluations over one week indicated full recovery with normalized prolactin levels. This case underscores the necessity of including lymphangioma in the differential diagnosis of young chest wall tumors. Although this condition is uncommon, early detection and appropriate surgical intervention result in favorable outcomes with a low chance of recurrence.

## Introduction

Lymphangiomas, also known as cystic hygromas, are congenital benign malformations of the lymphatic system. Their occurrence is due to obstruction of lymphatic pathways or the presence of sequestrated lymphatic sacs, resulting in the accumulation of lymph within cystically dilated channels that do not connect with other lymphatic capillaries or veins [1]. These lesions are hamartomatous proliferations of lymphatic vessels, predominantly diagnosed in childhood, with approximately 75% located in the neck and 20% in the axillary area [1,2]. Chest wall involvement is exceptionally rare, making diagnosis in this location more challenging [2].

Gynecomastia is a benign swelling in the male breast caused by glandular tissue proliferation. It is more common in infancy, adolescence, and middle-aged to elderly men. Prepubertal gynecomastia is uncommon and generally considered pathological, necessitating investigation for an underlying estrogen source. Although hyperestrogenemia can be endogenous or exogenous, the majority of patients with prepubertal gynecomastia have normal serum sex steroid concentrations, and no identifiable cause has been found [3]. Clinically, chest wall lymphatic malformations may mimic gynecomastia or other breast masses, and distinguishing between these conditions is essential to avoid misdiagnosis and ensure appropriate management [2].

Breast lymphangiomas are most commonly reported in the elderly, where they appear as slowly growing, painless masses that can be confused with other benign or malignant breast conditions. As a result, a comprehensive diagnostic evaluation involving imaging and laboratory techniques, as well as a clinical examination, is required [4].

This study describes a rare pediatric chest wall cystic hygroma that clinically simulated gynecomastia, highlighting the diagnostic challenges and surgical management considerations in this unusual presentation, in accordance with SCARE (Surgical CAse REport) guidelines [5].

## Case presentation

A three-year-old male presented with a rapidly growing mass in the right breast area. According to the family, the swelling initially presented as a small lump, encouraging them to try home treatment with topical creams and massage. The mass continued to increase in size rather than regress. The child had no relevant medical history of trauma, infection, or previous surgical interventions. He maintained excellent overall wellness, achieving normal growth and developmental milestones. The familial history revealed no hereditary diseases or lymphatic anomalies.

**Figure 1:**
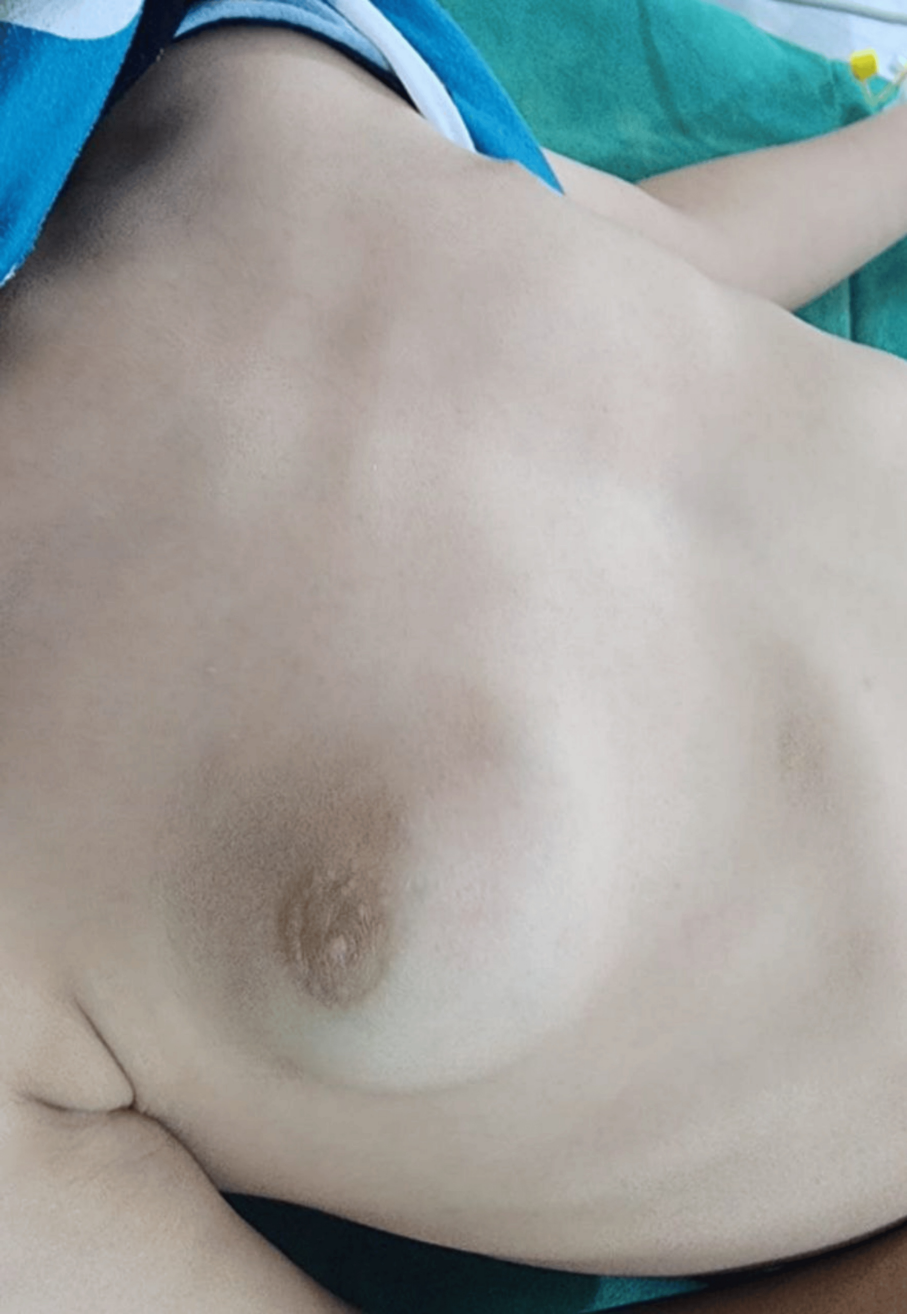
Preoperative clinical photograph showing a well-defined, unilateral breast swelling in a pediatric patient. The mass presents as a smooth, rounded prominence beneath the areola, with intact overlying skin and no visible erythema or ulceration.

On examination, a well-circumscribed, non-tender mass was palpated in the right chest wall at the breast region, measuring 3 x 4 cm, as seen in Figure 1. The lesion showed no signs of erythema, warmth, or changes in the overlying skin. An enlarged lymph node was found on the same side during examination. No additional masses were detected on the other side, and the remainder of the systemic examination was unremarkable.

Hormonal evaluation was performed to rule out endocrine causes of breast enlargement. The initial prolactin level was 17.4 ng/mL (normal pediatric range: 3.45-17.42 ng/mL for children under 12 years). A follow-up measurement showed mild elevation at 22 ng/mL, which normalized postoperatively at 12.5 ng/mL (normalized). Testosterone, estradiol, and progesterone levels were within normal ranges. We assumed that the transient prolactin elevation was attributed to breast massage, which can stimulate pituitary secretion.

**Figure 2:**
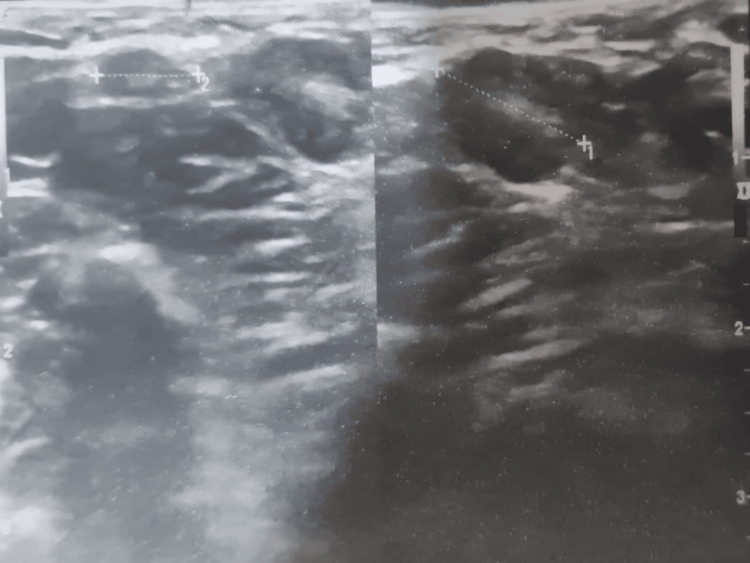
Ultrasound revealed multiloculated, hypoechoic cystic lesions with septa of varying thickness.

Ultrasonography (US) showed multiloculated, hypoechoic cystic lesions featuring linear septa of different thicknesses. The largest cyst was oval and measured 26 mm in diameter. There were no changes in mammary ducts, indicating the preservation of normal breast architecture. Lymphadenopathy was detected in the right axilla, with a diameter of 10 mm, as shown in Figure 2.

**Figure 3:**
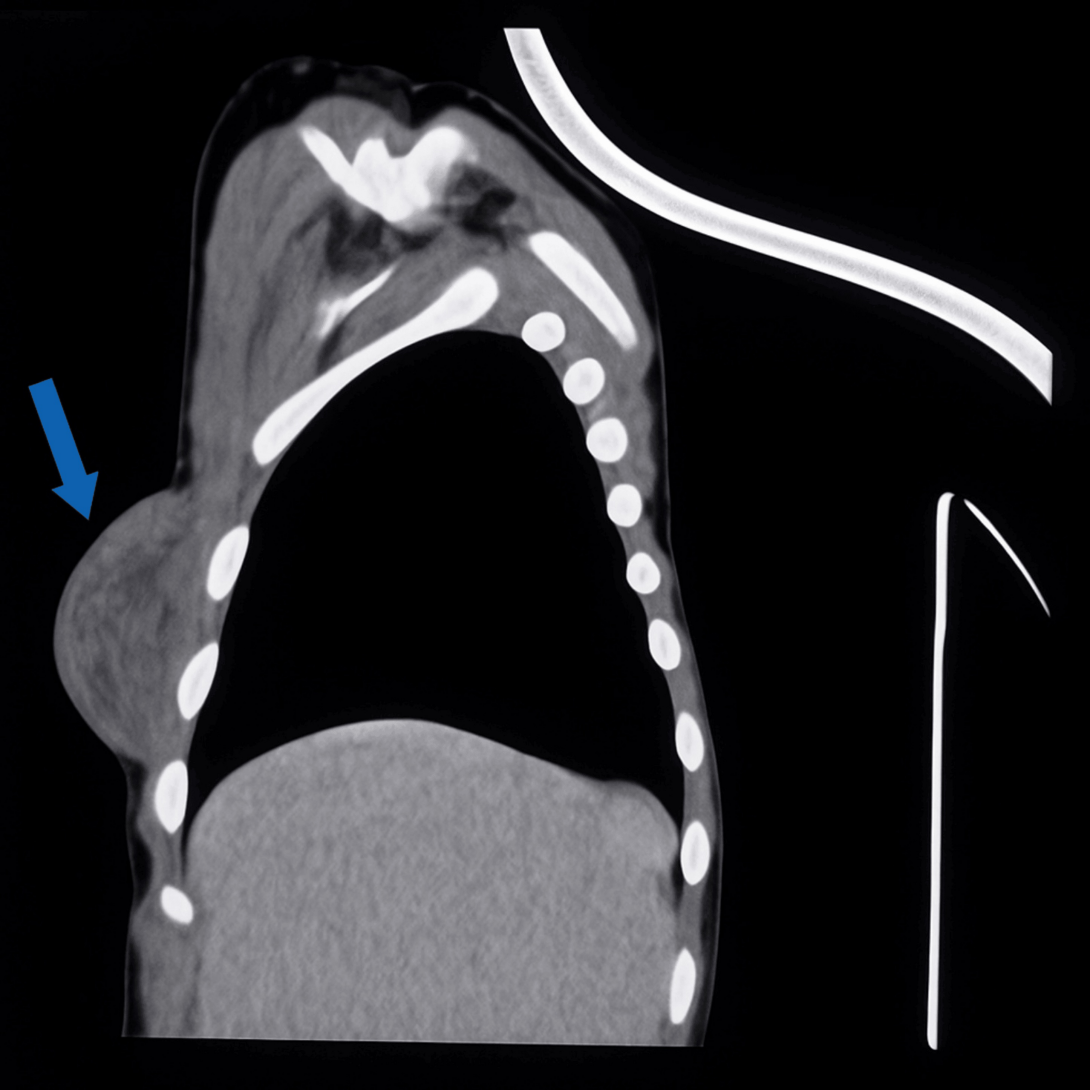
CT scan reveals a well-defined, encapsulated subcutaneous mass in the right chest wall.

Computed tomography (CT) of the thorax revealed normal cardiac and pulmonary parenchyma. The mass was in the right chest wall, lying subcutaneously above the muscular layer. The lesion was presented as a well-defined, encapsulated oval mass measuring 51 × 26 mm (Figure 3). Imaging features initially suggested a fibroma, although the differential diagnosis remained broad.

**Figure 4:**
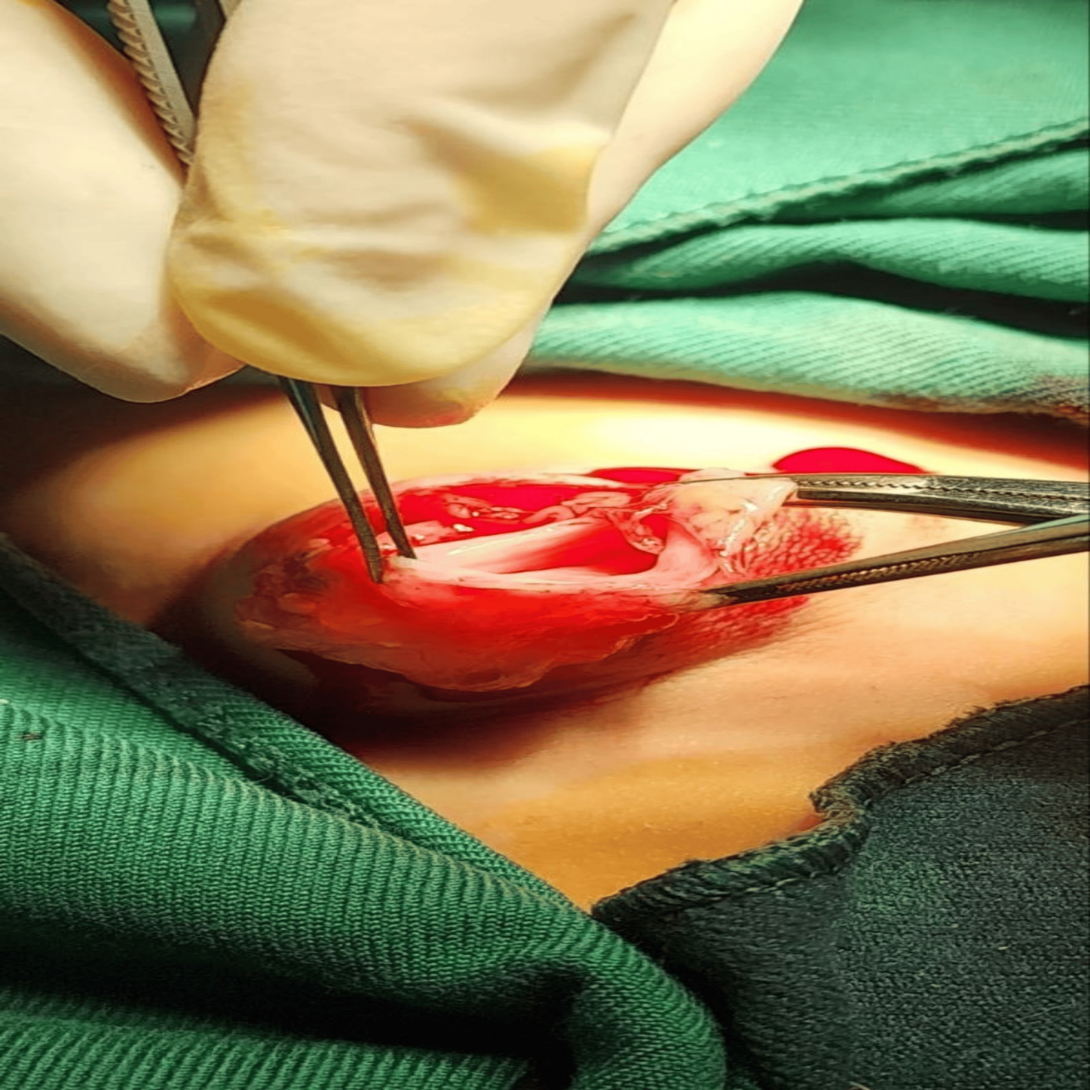
An intraoperative image showing a curvilinear incision was made over the palpable breast mass, following the natural skin crease to optimize cosmetic outcome. The dissection was carried carefully through the subcutaneous tissue to expose the lesion, showing a well-defined cyst containing translucent, gelatinous material.

As the mass continued to enlarge and its nature remained uncertain, we proceeded with surgical excision to obtain a definitive histopathological diagnosis. The procedure entailed the complete excision of the swelling while meticulously safeguarding the surrounding normal tissue structures. Intraoperative findings revealed a well-defined cystic structure containing translucent, gelatinous material, as shown in Figure 4.

The histopathological analysis revealed a well-defined gray-to-yellow mass measuring approximately 4.5 × 3.5 × 2 cm. The excised mass specimen consists of a well-defined cystic mass surrounded by fibroadipose tissue. Samples were collected from all regions for detailed microscopic analysis.

**Figure 5:**
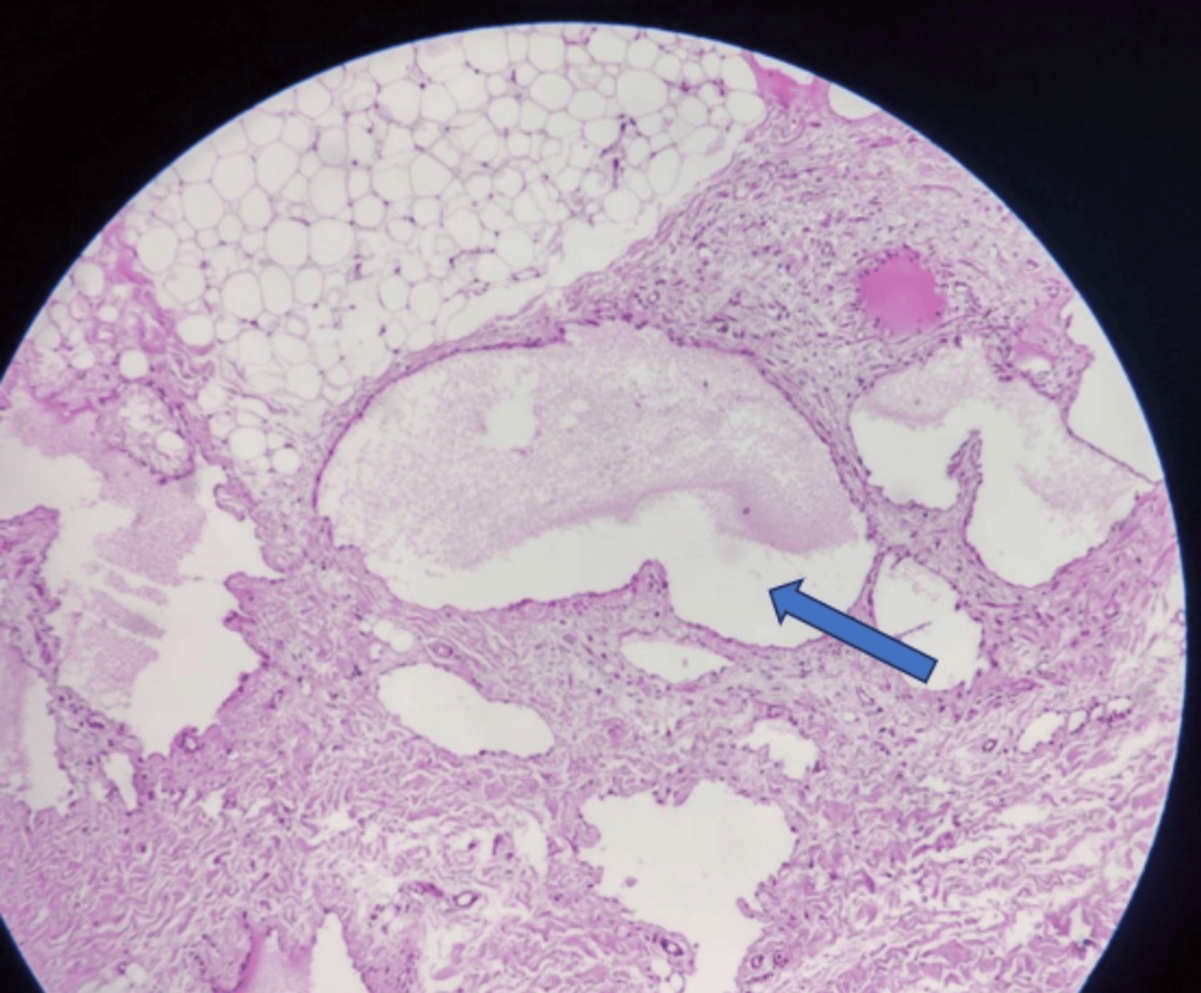
Microscopic specimen shows dilated, thin-walled lymphatic vessels with flattened endothelium. These channels are surrounded by focal fibrosis and scattered lymphocytes. Lumina contain eosinophilic proteinaceous fluid.

A microscopic examination revealed multiple lymphatic spaces forming a tumorous mass. These spaces were lined with flattened endothelial cells and lymphocytes from the stroma. Notably, there was little blood inside the lumens, distinguishing them from vascular malformations. Large vessels had poorly developed smooth muscle in their walls. The structure showed microscopically large lumens of cavernous and smaller capillary types, which are typical of lymphatic malformations. Importantly, no specific mammary tissue was found in the specimen, and the absence of malignancy confirmed the diagnosis of cystic hygroma of the chest wall in the right breast region, as illustrated in Figure 5.

The postoperative recovery was uneventful. A one-week follow-up exam revealed complete healing and no complications. The child's health was satisfactory, and the repeated prolactin level was normal at 12.5 ng/mL. After a month, there was no evidence of recurrence during follow-up.

## Discussion

While lymphangiomas are well-known benign lymphatic malformations, their presence in the breast or chest wall of children is uncommon. Gynecomastia in boys, whether unilateral or bilateral, may be caused by a familial increase in extraglandular aromatization of the androgen mechanism, as well as other known causes such as hormonal changes, malignancy, liver disease, obesity, or specific drugs [6]. The most common mechanism is estrogen overproduction, which can be caused by direct secretion, decreased clearance, increased aromatization, or exogenous supply [7].

Our case is a rare example of male unilateral gynecomastia caused by breast lymphangioma. There have been numerous reports of gynecomastia in prepubertal age due to various causes, including cystic hygroma. For example, Kaye and Leddy found 17 cases of breast lymphatic abnormalities in the medical literature, with only four cases in children and one unusual case in a young female patient [8]. Al-Salem's investigation of 22 children with lymphatic malformations revealed that only one child, a one-year-old girl, had breast lymphatic malformation, characterized by left breast enlargement, which was successfully treated with surgical excision and histological confirmation of lymphangioma [1]. Gupta and Singh reported on an eight-year-old male with cystic lymphangioma of the breast who received complete excision as treatment [9]. Furthermore, Singh et al. and Ekmez et al. reported cases similar to ours in terms of age demographics, clinical manifestations, and successful surgical interventions [4,10].

In our case, we assumed that the progressive enlargement of the mass was caused by massaging the area and stimulating the mammary glands, which resulted in the release of prolactin as a natural physiological change. Prolactin is the primary regulatory hormone for mammary gland development and function. It promotes the growth of mammary alveoli, the mammary gland's milk-producing components, and stimulates breast alveolar epithelial cells to produce essential milk components, such as proteins, carbohydrates, and lipids. Prolactin is important for mammary morphogenesis at different stages of development. It controls ductal side branching, terminal end bud regression, and lobuloalveolar development [11]. Massage of the breast is known to stimulate prolactin release by mechanically stimulating sensory receptors in breast tissue, activating nerve impulses transmitted through the spinal cord to the hypothalamus. This neural pathway reduces prolactin-inhibiting factor (dopamine), removing its inhibitory effect on lactotrophs [12]. Another theory suggests that breast massage increases oxytocin release and prolactin-mediated responses. Direct massage of the breast area does not produce a surge of prolactin in the same way that suckling does; however, it does stimulate hormone production via tactile stimulation pathways [13].

Cystic hygroma must be diagnosed through a comprehensive assessment that includes highly sensitive imaging modalities such as magnetic resonance imaging (MRI) and ultrasound, as well as a histopathological examination of the mass. While mammography, ultrasound, and MRI can help with diagnosis, they may not always provide conclusive results for cystic hygroma. On mammography, these lesions appear as thick, lobulated masses that can be localized or diffuse and are usually free of micro- or macrocalcifications. In the US, multiple septated cystic formations with clear fluid inside can be seen, as well as low-level internal echoes indicative of hemorrhage or proteinaceous material [14].

While complete surgical excision remains the gold standard, various options have been documented based on the depth, location, patient age, and overall health. These include conservative observation, surgery, sclerotherapy, and medical management, with surgical excision and sclerotherapy considered better options with cosmetic outcomes [15,16]. Kaye and Leddy reported an adolescent case that demonstrated the use of sclerotherapy with doxycycline as the first line of treatment, followed by sirolimus therapy for recurring disease [8]. However, the lesion's complex, infiltrative nature and proximity to vital structures required these alternative approaches [8].

## Conclusions

This study presents a case of a rare pediatric cystic hygroma, marked by progressive growth and specific lymphatic anomalies. It underscores the necessity of incorporating lymphatic malformations in the differential diagnosis of pediatric breast and chest wall masses, using ultrasound as the principal imaging technique and histopathologic examination as the definitive diagnostic criterion. The clinical progression indicates that repeated local manipulation may have induced temporary endocrine alterations, while total surgical excision led to an uncomplicated recovery. This case enhances clinical awareness of this atypical localization and illustrates the value of early recognition and appropriate management in pediatric care.

## References

[1] Al-Salem AH (2004). Lymphangiomas in infancy and childhood. Saudi Med J.

[2] Minocha PK, Roop L, Persad R (2014). Cases of atypical lymphangiomas in children. Case Rep Pediatr.

[3] Arya R, Rathi AK, Singh K (2016). Gynecomastia: a review of literature. MAMC J Med Sci.

[4] Singh N, Kumari G, Singh DK (2025). Imaging features of breast lymphangioma: a rare case report. Radiol Case Rep.

[5] Sohrabi C, Mathew G, Maria N, Kerwan A, Franchi T, Agha RA (2023). The SCARE 2023 guideline: updating consensus Surgical CAse REport (SCARE) guidelines. Int J Surg.

[6] Hoevenaren IA, Schott DA, Otten BJ, Kroese-Deutman HC (2011). Prepubertal unilateral gynecomastia: a report of two cases. Eur J Plast Surg.

[7] Sansone A, Romanelli F, Sansone M, Lenzi A, Di Luigi L (2017). Gynecomastia and hormones. Endocrine.

[8] Kaye R, Leddy R (2022). Pediatric breast lymphatic malformation with recurrent presentation in an adolescent female. BJR Case Rep.

[9] Gupta SS, Singh O (2011). Cystic lymphangioma of the breast in an 8-year-old boy: report of a case with a review of the literature. Surg Today.

[10] Ekmez F, Pirgon O, Bilgin H, Aydemir G (2012). Cystic hygroma of the breast in a 5 year old boy presenting as a gynecomastia. Eur Rev Med Pharmacol Sci.

[11] Brisken C, Kaur S, Chavarria TE (1999). Prolactin controls mammary gland development via direct and indirect mechanisms. Dev Biol.

[12] Al-Chalabi M, Bass AN, Alsalman I (2025). Physiology, prolactin. StatPearls.

[13] Yokoyama Y, Ueda T, Irahara M, Aono T (1994). Releases of oxytocin and prolactin during breast massage and suckling in puerperal women. Eur J Obstet Gynecol Reprod Biol.

[14] Agarwal S, Doctor M, Ruidas S, Lal H (2020). Cystic lymphangioma of breast: a rare presentation. BMJ Case Rep.

[15] Waner M, O TM (2018). Multidisciplinary approach to the management of lymphatic malformations of the head and neck. Otolaryngol Clin North Am.

[16] McCaffrey F, Taddeo J (2015). Surgical management of adult-onset cystic hygroma in the axilla. Int J Surg Case Rep.

